# Pesticides reduce tropical amphibian and reptile diversity in agricultural landscapes in Indonesia

**DOI:** 10.7717/peerj.15046

**Published:** 2023-03-20

**Authors:** Thomas Cherico Wanger, Barry W. Brook, Theodore Evans, Teja Tscharntke

**Affiliations:** 1 Agroecology, University of Göttingen, Göttingen, Germany; 2 Sustainable Agricultural Systems & Engineering Laboratory/School of Engineering, Westlake University, Hangzhou, China; 3 University of Tasmania, Hobart, Australia; 4 University of Western Australia, Perth, Australia; 5 Department of Biological Sciences, National University of Singapore, Singapore, Singapore

**Keywords:** Cypermethrin, Glyphosate, Indonesia, Integrated pest management, Paraquat, Pest control, Pesticides, Indirect effects

## Abstract

Pesticide use on tropical crops has increased substantially in recent decades, posing a threat to biodiversity and ecosystem services. Amphibians and reptiles are common in tropical agricultural landscapes, but few field studies measure pesticide impacts on these taxa. Here we combine 1-year of correlative data with an experimental field approach from Indonesia. We show that while pesticide application cannot predict amphibian or reptile diversity patterns in cocoa plantations, our experimental exposure to herbicides and insecticides in vegetable gardens eliminated amphibians, whereas reptiles were less impacted by insecticide and not affected by herbicide exposure. The pesticide-driven loss of a common amphibian species known to be a pest-control agent (mainly invertebrate predation) suggests a strong indirect negative effect of pesticides on this service. We recommend landscape-based Integrated Pest Management and additional ecotoxicological studies on amphibians and reptiles to underpin a regulatory framework and to assure recognition and protection of their ecosystem services.

## Introduction

The continuing growth of the human population requires an increase in agricultural productivity to secure the rising demand for food, fibre and bioenergy. This demand has led to agricultural expansion, mainly in the tropics, which has led to subsequent biodiversity loss (Foley et al., 2011). Consequently, there is an ongoing debate about how agricultural production in the tropics can produce higher yields and yet also protect biodiversity (Wanger et al., 2020; Tamburini et al., 2020). Some argue that intensifying production on existing agricultural land will allow conservation of biodiverse areas (‘land sparing’), while others contend that conservation value of agricultural land can be improved to protect biodiversity and ecosystem services (‘land sharing’; Grass et al., 2019). In practice, the decision will typically depend on the specific location and species groups (Kremen, 2015). For example, amphibians and reptiles are threatened by land-use change, but some species appear to survive well under land-sharing scenarios (Wanger et al., 2010a).

Land-sharing will result in pesticide exposure for amphibians and reptiles with negative impacts known primarily from laboratory studies for herbicides such as atrazine and glyphosate and the insecticide endosulfan (Berger, Graef & Pfeffer, 2013; Grant, Woudneh & Ross, 2013; Hayes et al., 2010; Relyea, Schoeppner & Hoverman, 2005). These substances are mostly banned in the European Union (EU) but are still commonly used in tropical countries, where little is known about the effect of increasing pesticide application on amphibians and reptiles (Wanger, Rauf & Schwarze, 2010). A review investigated the effects of pesticides on amphibians and reptiles based on 1,336 studies and 23,152 comparisons (Ortiz-Santaliestra et al., 2017), and showed that species exposed in mesocosm studies were 1,429 times more sensitive than in field experiments when lethal effect were considered. However, information about pesticide toxicity for terrestrial amphibians and reptiles in general is limited (but see Brühl et al., 2013; Agostini et al., 2020) and, hence, also precludes extrapolating results from other terrestrial taxa such as birds and mammals (Ortiz-Santaliestra et al., 2017). Given the lack of knowledge from European and North American systems, it is not surprising that this pattern is exacerbated in a tropical context. To the best of our knowledge, long-term data and field experiments to predict effects of pesticide application on tropical amphibians and reptiles in agricultural landscapes are missing.

Addressing this data gap, we built on a unique opportunity in cocoa production landscapes in Indonesia, where pesticide use started at the onset of our study. We combine a correlative field survey across five habitat types (primary forest, secondary forest, cacao agroforests, cacao monocultures, and clear-cut grassy areas) to evaluate how pesticide use affects amphibians and reptiles on the plot and landscape scale in Sulawesi, Indonesia. Pesticide use on the landscape scale may prevent non-target organisms from avoiding pesticide exposure and will affect food sources. We also experimentally exposed amphibians and reptiles to realistic pesticide application patterns in the field. The combined correlative-experimental field approach and our two-taxon comparison allowed a realistic assessment of the effects of agricultural intensification on biodiversity and the implications for ecosystem-service provisioning. We tested the hypothesis that both, amphibian and reptile richness and abundance will be measurably affected by commonly applied insecticides and herbicides.

## Methods

### Study site

We conducted our study in Central Sulawesi (Indonesia) around Lore Lindu National Park (231,000 ha of pristine forest), where farming activities and pesticide use have increased over the past years (Wanger, Rauf & Schwarze, 2010). Mean annual temperature and precipitation are 24.0 (±0.16 SD) °C and 1,437 (±227.4 SD) mm, respectively. We did the correlative surveys near the village Toro (1°30′24″S, 120°02′11″E) and the experiments in the Napu valley (1°25′20″S 120°18′44″E; for details see (Motzke et al., 2013; Wanger et al., 2010a); and Fig. S4). We targeted five habitat types: primary forest, secondary forest, cacao agroforests, cacao monocultures, and grassy clearings (hereafter ‘open areas’),

### Correlative approach

To determine long-term pesticide use in the study region, we distributed a standardized questionnaire to all owners of agricultural plots (*n* = 36) before the first and after the last amphibian and reptile sampling session. We identified the pesticide brand used and the number of pesticide container caps applied per month per plot, because this was an easier measure for the farmers to report amounts. From the original pesticide containers, we then recorded the amount of pesticide per cap, the amount of caps per 10 liters of water, and dosage instructions. While there is a risk that the respondents provide false information, we argue that these effects are limited in our case. This is because (i) we saw the pesticide containers used and (ii) asked the plot owners to show us with the actual amounts how they are preparing the tanks for pesticide use. All plot owners gave their oral and informed consent to voluntarily participate in the questionnaire. We then calculated our plot -scale variables: the amount of pesticide applied per application (the variable *amo*); the frequency of pesticide application/ plot/year (*freq*); and the total amount of pesticide applied/ plot/ year (*t_amt*). For landscape-scale information, we asked the landowners of the areas directly surrounding our plots (*e.g.*, other cocoa plantations, open areas, *etc*.) if they were using pesticides (*surr*). *Surr* is a binary (yes/no) variable. Plot owners also stated that pesticide use before the sampling was negligible. For additional details see Wanger et al. (2010a).

To determine biodiversity metrics, we sampled amphibians and reptiles on 31 × 0.16 ha plots (40 × 40 m) with acoustic and visual encounter surveys. Specifically, we sampled 2 × 56.5 m diagonal cross-transects per plot within five habitat types, *i.e.,* primary forest (*n* = 6), secondary forest (*n* = 7), natural-shade cocoa (*n* = 7), planted-shade cocoa (*n* = 6), and open areas (*n* = 5). All plots were located *in situ* within the same habitat type and at least 1,000 m apart from each other to avoid spatial autocorrelation. We toe-clipped all individuals captured to avoid double counting. We did not mark the captured snakes, because the abundances were low (*n* = 1). In case of *Ahaetulla prasina* (*n* = 2), we did not encounter the same species in the same or adjacent plots. The first sampling session was completed in December 2007 and the last in July 2008. In total, we conducted 186 sampling sessions on the 31 plots in two sessions, six times per plot, three times during the day (6.00 h to 18.00 h) and three times during the night (18.00 h to 6.00 h). Sampling on the transects was restricted to 25 min (for details on the sampling regime and habitat descriptions, see Wanger et al., 2010a and Table S1). This study differs from our previous work in that it contains additional data with a focus on pesticide effects on amphibians and reptiles.

### Experimental approach

The experiment consisted of 15 × 0.03 ha plots (17.5 × 17.5 m) with a 1,000 m minimum distance between plots. Each was planted with four vegetable species (carrot, cucumber, aubergine, and chili) to resemble a typical vegetable cropping system. The split-plot design with four subplots (8.75 × 8.75 m each containing all vegetable species) per plot contained one of the following treatments: manual weeding, herbicide treatment (paraquat-dichlorid 297 g/L), insecticide treatment ( *α*-cypermethrin 30 g/L), and a combined herbicide/ insecticide treatment with concentrations applied to 0.008ha. We applied the pesticide treatment weekly in accordance with local management practices (Motzke et al., 2013). For substance details see Note S1. We sampled amphibians and reptiles following the methods above before pesticide application started and after when it had stopped, three months later.

### Analyses

For the correlative approach, we used Bayesian generalised linear mixed effects models with total amphibian and reptile richness (*aspr* and *rspr*) and abundance (*aabd* and *rabd*) per plot, and separately, the abundance of the most common amphibian (*Ingerophrynus celebensis* [*ic*] and *Hylarana celebensis* [*hc*]) and reptile species (*Eutropis multifasciatus* [*em*]), as response variables. We did consider completeness of sampling effort and used species accumulation curves and bootstrapping to evaluate errors in species richness estimates per plot. However, we did not find errors in richness estimators and, hence, used original data for our modelling here (see Wanger et al., 2010a). The common species *ic* and *hc* were used because common species are more robust towards human disturbance (Wanger et al., 2010a; Wanger et al., 2011) and—if we find an effect on these species—rare and more sensitive species are likely affected more severely (for a species list see Tables S2A and S2B). We included plot- (*t_amt*, *freq*, *amo*) and landscape-level explanatory variables (*surr*). We tested for the most suitable random effect structure between sampling habitat (*hab*) and plot id (*plotID*) to account for the aggregate of idiosyncratic habitat-specific environmental parameters not captured by the treatment predictors (Wanger et al., 2010a; Wanger et al., 2009; Tables 1 and 2). We did not incorporate these environmental parameters individually in our model set to avoid model-variant saturation. We also tested the most suitable distribution of our models between Poisson, negative binomial (to account for overdispersion), and accounting for zero inflation for both distributions (see Zuur et al. (2009) and Tables 1 and 2 for details).

**Table 1:** Pesticide-specific determinants of amphibian species richness and overall abundance (top two sub-tables) and abundance of the two most common species (bottom two sub-tables).

**Amphibian Species Richness**				
**re = hab; zip; qr decomposition**	**elpd**_**loo**_ **± SE**	**Δelpd ± SE**	**p**_**loo**_ **± SE**	**loo**_**ic**_ **± SE**
***aspr ∼ freq***	**−51.77 ± 6.79**	**0.00 ± 0.00**	**4.01 ± 0.93**	**103.55 ± 13.58**
***aspr ∼ freq + year***	**−52.06 ± 6.77**	**−0.28 ± 1.10**	**4.61 ± 1.00**	**104.11 ± 13.54**
***Null***	**−52.36 ± 6.90**	**−0.59 ± 1.96**	**4.31 ± 1.06**	**104.73 ± 13.79**
*aspr ∼ surr*	−53.15 ± 7.02	−1.38 ± 1.92	4.83 ± 1.12	106.31 ± 14.05
*aspr ∼ freq + year + surr + amo*	−53.31 ± 7.18	−1.54 ± 1.38	6.99 ± 1.52	106.62 ± 14.36
*aspr ∼ amo*	−53.37 ± 6.93	−1.60 ± 1.05	4.81 ± 1.13	106.75 ± 13.87
**Amphibian Abundance**				
**re = hab; zinb; qr decomposition**	**elpd**_**loo**_ **± SE**	**Δelpd ± SE**	**p**_**loo**_ **± SE**	**loo**_**ic**_ **± SE**
***aabd ∼ freq***	**−66.17 ± 7.19**	**0.00 ± 0.00**	**4.58 ± 0.83**	**132.34 ± 14.37**
***Null***	**−66.58 ± 7.21**	**−0.42 ± 1.43**	**4.50 ± 0.79**	**133.17 ± 14.42**
*aabd ∼ amo*	−66.89 ± 7.19	−0.73 ± 1.08	4.89 ± 0.85	133.79 ± 14.38
*aabd ∼ freq + year*	−67.13 ± 7.24	−0.96 ± 0.57	5.49 ± 0.89	134.26 ± 14.48
*aabd ∼ surr*	−67.61 ± 7.29	−1.44 ± 1.48	4.66 ± 0.81	135.22 ± 14.57
***Ingerophrynus celebensis*** **Abundance**				
**re = plot id; zinb; qr decomposition**	**elpd**_**loo**_ **± SE**	**Δelpd ± SE**	**p**_**loo**_ **± SE**	**loo**_**ic**_ **± SE**
***Null***	**−40.22 ± 7.59**	**0.00 ± 0.00**	**4.59 ± 1.78**	**80.45 ± 15.20**
*ic ∼ surr*	−41.08 ± 7.66	−0.86 ± 0.66	5.39 ± 1.81	82.18 ± 15.32
*ic ∼ amo*	−41.25 ± 7.75	−1.03 ± 0.90	5.54 ± 1.88	82.52 ± 15.52
*ic ∼ freq*	−41.46 ± 7.75	−1.24 ± 0.48	5.55 ± 1.91	82.92 ± 15.52
***Hylarana celebensis*** **Abundance**				
**re = hab; nb; qr decomposition**	**elpd**_**loo**_ **± SE**	**Δelpd ± SE**	**p**_**loo**_ **± SE**	**loo**_**ic**_ **± SE**
***Null***	**−33.62 ± 8.27**	**0.00 ± 0.00**	**3.48 ± 1.14**	**67.24 ± 16.55**
*hc ∼ freq*	−34.93 ± 8.43	−1.32 ± 1.59	4.38 ± 1.45	69.87 ± 16.88
*hc ∼ amo*	−35.12 ± 8.57	−1.50 ± 1.56	4.49 ± 1.59	70.24 ± 17.15
*hc ∼ surr*	−35.37 ± 8.58	−1.75 ± 2.18	4.66 ± 1.75	70.74 ± 17.18

**Notes.**

Shown are all models with a Δelpd <2 and the null model.

Abbreviations Null null model including random effect and intercept (*interc*) term aspr amphibian species richness aabd amphibian abundance ic *Ingerophrynus celebensis* abundance hc *Hylarana celebensis* abundance t_amt total amount of pesticides applied/plot/year amo pesticide amount applied/application freq herbicide application frequency/year surr pesticide use in the surrounding cocoa plantations *elpd*_*loo*_ Bayesian leave-one-out (LOO) estimate of out-of-sample predictive fit. The expected log pointwise predictive density (*espd*) is a measure of predictive accuracy that is comparable and interpretable across different scales of effective number of parameters in the dataset Δ*elpd* is the difference between the best fitting model and the focus model *p*_*loo*_ effective number of parameters *loo*_*ic*_ −2 *elpd*_*loo*_

Rows in bold indicate the null model and all models that are ranked higher than the null model based on delta elpd.

**Table 2:** Pesticide-specific determinants of reptile species richness and overall abundance (top two sub-tables) and abundance of the most common species (bottom sub-table).

**Reptile Species Richness**				
**re = plot id; zinb; qr decomposition**	**elpd**_**loo**_ **± SE**	**Δelpd ± SE**	**p**_**loo**_ **± SE**	**loo**_**ic**_ **± SE**
***Null***	**−64.49 ± 8.32**	**0.00 ± 0.00**	**11.56 ± 2.17**	**128.98 ± 16.66**
*rspr ∼ amo*	−64.76 ± 8.33	−0.27 ± 0.97	12.69 ± 2.27	129.52 ± 16.66
*rspr ∼ freq*	−64.87 ± 8.48	−0.38 ± 0.84	13.10 ± 2.39	129.74 ± 16.96
*rspr ∼ surr*	−64.95 ± 8.38	−0.46 ± 0.71	12.49 ± 2.18	129.89 ± 16.76
*rspr ∼ freq + year*	−66.31 ± 8.68	−1.82 ± 1.03	13.84 ± 2.51	132.63 ± 17.35
**Reptile Abundance**				
**re = plot id; zinb**	**elpd**_**loo**_ **± SE**	**Δelpd ± SE**	**p**_**loo**_ **± SE**	**loo**_**ic**_ **± SE**
***Null***	**−85.96 ± 6.50**	**0.00 ± 0.00**	**11.74 ± 1.44**	**171.93 ± 13.00**
*rabd ∼ surr*	−86.29 ± 6.61	−0.32 ± 0.79	12.91 ± 1.48	172.57 ± 13.23
*rabd ∼ amo*	−86.39 ± 6.47	−0.43 ± 0.36	12.32 ± 1.40	172.78 ± 12.94
*rabd ∼ freq*	−86.76 ± 6.61	−0.79 ± 0.32	12.83 ± 1.48	173.51 ± 13.23
*rabd ∼ freq + year + surr + amo*	−87.54 ± 6.83	−1.58 ± 1.49	14.96 ± 1.68	175.09 ± 13.66
***Eutropis multifasciatus*** **Abundance**				
**re = hab; zip; qr decomposition**	**elpd**_**loo**_ **± SE**	**Δelpd ± SE**	**p**_**loo**_ **± SE**	**loo**_**ic**_ **± SE**
***Null***	**−25.48 ± 7.44**	**0.00 ± 0.00**	**3.71 ± 1.52**	**50.97 ± 14.89**
*em ∼ surr*	−25.52 ± 7.38	−0.04 ± 0.47	3.70 ± 1.48	51.05 ± 14.75
*em ∼ freq*	−26.63 ± 7.69	−1.15 ± 0.81	4.83 ± 1.85	53.26 ± 15.38
*em ∼ amo*	−26.85 ± 7.94	−1.36 ± 0.61	5.03 ± 2.09	53.69 ± 15.89

**Notes.**

Shown are all models with a Δelpd <2 and the null model.

Abbreviations Null null model including random effect and intercept (*interc*) term rspr reptile species richness rabd reptile abundance em *Eutropis multifasciatus* abundance t_amt total amount of pesticides applied/plot/year amo pesticide amount applied/application freq herbicide application frequency/year surr pesticide use in the surrounding cocoa plantations *elpd*_*loo*_ Bayesian leave-one-out (LOO) estimate of out-of-sample predictive fit. The expected log pointwise predictive density (*espd*) is a measure of predictive accuracy that is comparable and interpretable across different scales of effective number of parameters in the dataset Δ*elpd* is the difference between the best fitting model and the focus model *p*_*loo*_ effective number of parameters *loo*_*ic*_ −2 *elpd*_*loo*_

Rows in bold indicate the null model and all models that are ranked higher than the null model based on delta elpd.

For all model checks including the most appropriate random effect structure, model distribution, and best model fit, we used Pareto Smoothed Importance Sampling leave-one-out cross validation (PSIS-LOO) instead of information criteria (*e.g.*, the Deviance Information Criterion–DIC; or the more robust Watanabe-Aikaike Information Criterion–WAIC). Although WAIC estimates are similar to PSIS-LOO, the latter is more robust in cases of weakly informative priors or influential observations (for a detailed and technical discussion on WAIC and PSIS-LOO comparisons see Vehtari, Gelman & Gabry, 2017). The shape parameter khat of the pareto distribution can be used to assess the reliability of the leave-one-out cross validation (LOO) estimates (Vehtari, Gelman & Gabry, 2017). We used expected log pointwise predictive density (*elpd*) for each model as a measure of predictive accuracy that is comparable and interpretable across different scales of effective number of parameters in a dataset (Vehtari, Gelman & Gabry, 2017). For multi-model inference we considered only those model subsets that did not contain a combination of *t_amt with amo*, and *freq* to avoid collinearity problems. We also used QR decomposition to further reduce collinearity when fitting the model (see Tables 1 and 2). We used posterior predictive checks for all models to understand whether the best models can predict maximum and minimum values of the observed data. We used the loo (Vehtari, Gelman & Gabry, 2017) and brms (Bürkner, 2017) packages in the R software (v.4.2.2; http://www.r-project.org) for this analysis.

For the experimental approach, we used Bayesian regression modelling to measure the individual and additive effects of pesticide treatments on amphibian and reptile species richness and abundance, and the most abundant amphibian (*I. celebensis*) and reptile species (*Eutropis* spp.). As the response variable, we used the difference between the two richness and abundance sampling sessions, to reduce the number of estimated parameters in the model. In the generalised linear mixed effects model structure we included plots (*plot*) as a random effect to account for plot-specific differences and repeated sampling that are not of interest relative to the modelled treatment effect. We nested treatment (*treat*) as a fixed effect within *plot* to account for treatments in individual plots, and added the landscape-level variable distance to forest (*dist*) as a second fixed effect. After multi-model inference, the final model structure included *plot* and *treat* (nested within *plot*) as respective random and fixed effects. We performed this analysis in the Program R v2.13 ( http://www.r-project.org) using the packages R2WinBugs (Sturtz, Ligges & Gelman, 2005) and glmmADMB (Fournier et al. , 2012).

We thank the Indonesian Ministry of Research and Technology (RISTEK) and the Indonesian Institute of Sciences (LIPI) for granting permission to conduct this study for issuing the research permit (0048/EXT/SIP/FRP/SM/X/2010, 1899/FRP/SM/VIII/2008, and 7374a/SU/KS/2007). All methods including animal handling and human participants were carried out in accordance with relevant guidelines and regulations by RISTEK and LIPI.

## Results

The pesticide survey to determine long-term pesticide use revealed that the Indonesian farmers used herbicides and not insecticides on the 36 study plots for the correlative approach (note that this is not the case in the experimental study area where insecticides are regularly applied; Motzke et al., 2013). Specifically, they applied combinations of the herbicides glyphosate and paraquat (three owners used glyphosate, five owners used paraquat, six owners used both). Herbicide use across habitats in the study area increased by more than six-fold, from an average use of 0.3 litres per hectare (l/ha) to 2 l/ha of the same dosage, from 2007 to 2008 (see Fig. S1). Our first sampling in 2007 was able to serve as a temporal control, because (i) pesticide use in all habitats but clear cut grassy areas was close to zero; and (ii) locals reported that pesticide use was negligible before sampling started in the study area. Overall, we recorded 12 amphibian and 15 reptile species (Tables S2A and S2B).

The correlative approach showed that plot-level variables were the best predictors of amphibian richness and abundance in cocoa agroforestry landscapes (Table 1). Amphibian richness (*intercept* = −0.55 ± 0.48; *freq* = −0.38 ± 0.20 reported are mean coefficients and SE) and abundance decreased with an increasing herbicide application frequency (*intercept* = −0.13 ± 0.55; *freq* = −0.32 ± 0.16; Fig. S2B). However, these effects are not statistically robust, because the null model also ranked amongst the best models (Δ*elpd* < 4; Sivula, Magnusson & Vehtari, 2020; Table 1). Abundance of the generalist species, *Ingerophrynus celebensis* and the common forest species, *Hylarana celebensis*, was not predicted by either plot- or landscape-level predictors (null model was the model with the lowest *edpd*_*loo*_; Table 1). As a noteworthy observation, we found many *I. celebensis* and *Fejervarya limnocharis* (Fig. S2A) with deformed limbs in the intensively sprayed plots of our study area. By contrast, neither reptile species richness nor general or *Eutropis multifasciatus* abundance was predicted by plot- or landscape-level variables (Table 2; Fig. S2C).

**Figure 1:**
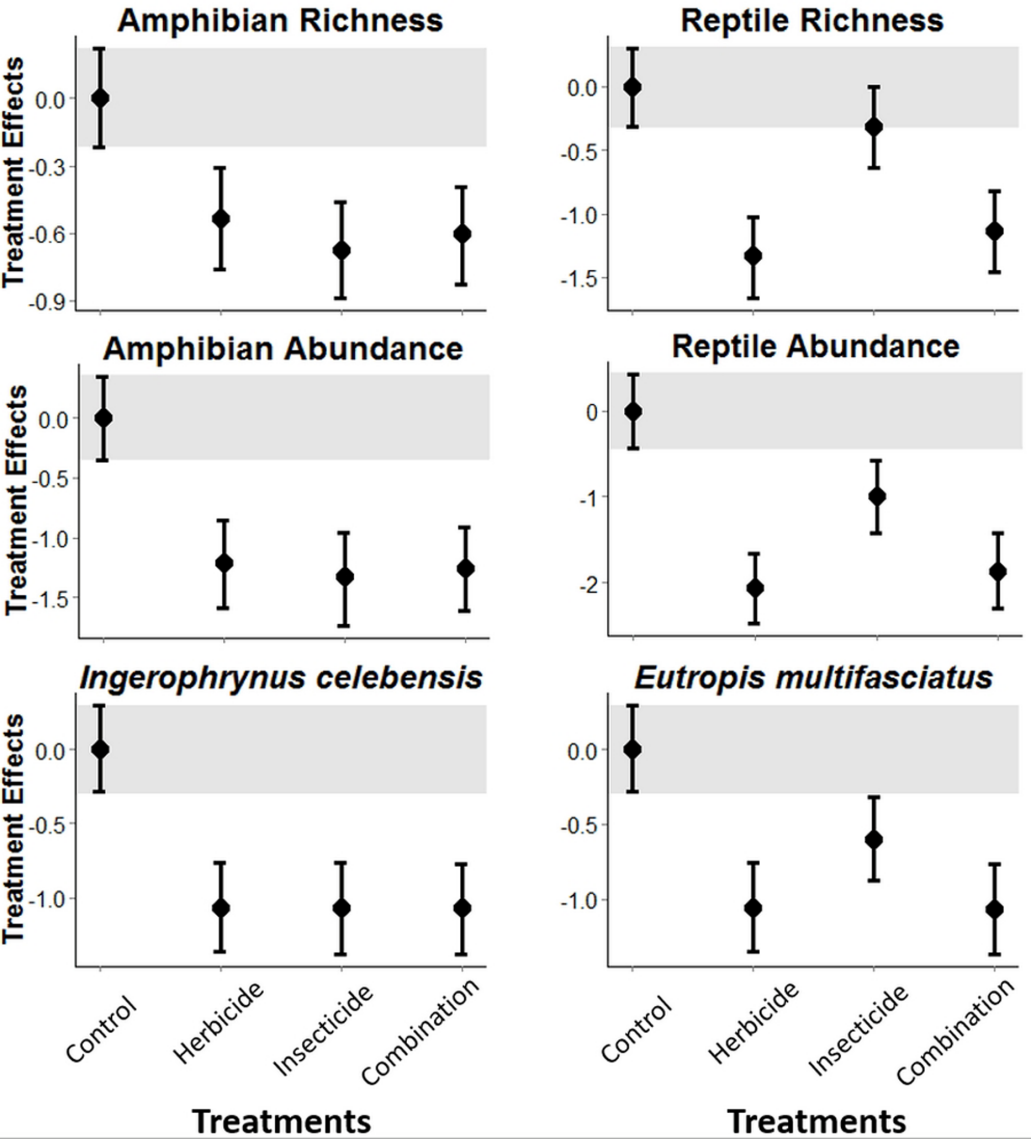
Pesticide treatment effects on amphibian and reptile richness and overall abundance, and abundance for the most common species for each of these taxonomic groups. A statistically measurable effect was found if the credibility intervals of the control (indicated through a grey bar) do not overlap with the error bars of the treatments.

The experimental application of pesticides in Indonesian vegetable plantations was detrimental to both amphibians and reptiles. Amphibian richness and abundance, and abundance of the *I. celebensis* decreased in all treatments compared with the control (*i.e.,* Bayesian credibility intervals of the control and treatment estimates do not overlap; Fig. 1 and Fig. S3). *I. celebensis* is the same species that was identified as a pest control agent of invasive ants in the same study area (Wanger et al., 2010b). The herbicide and the combined treatments—but not the insecticide—led to reduced reptile species richness compared with the control. All pesticide treatments reduced total reptile abundance, and the abundance of the common reptile species *Eutropis* spp. compared with the control (Fig. 1 and Fig. S3).

## Discussion

Combining 1-year field observations and a field experiment, we found a strong negative response of tropical amphibians and reptiles to pesticide use in tropical agricultural landscapes in Indonesia. Amphibians showed higher sensitivity to pesticide exposure than reptiles.

In this study, amphibians were exposed to the herbicides glyphosate and paraquat, and the insecticide cypermethrin. Most amphibian ecotoxicological data on glyphosate, the world’s most widely used herbicide, is from aquatic rather than terrestrial life stages (for a review see Wagner et al., 2013), including mixture effects with other pesticides (Annett, Habibi & Hontela, 2014; Lanctôt et al., 2014). Glyphosate can lead to altered developmental rates, physical malformations, and premature death (Lanctôt et al., 2014; Mann et al., 2009). Laboratoy studies have shown that paraquat is lethal to tadpoles, suppresses reproductive success, and is of high genotoxicity (Quassinti et al., 2009; Yin et al., 2008). Information on cypermethrin (insecticide) effects on tropical amphibians is scarce, but it acts synergistically with glyphosate further decreasing tadpole survival (Brodeur, Poliserpi & Sánchez, 2014).

Our results also showed that herbicide (*i.e.,* a mix of glyphosate and paraquat) application decreased amphibian species richness at the plot scale (although it also led to decreased abundance). The richness decrease might have resulted from a removal of sensitive specialist species such as *Limnonectes* spp. from the community. Although not statistically robust, the negative effect of application frequency on amphibian abundance corresponded with reported glyphosate impacts on amphibians (Lanctôt et al., 2014; Mann et al., 2009). The lack of detectable 1-year effects on the *I. celebensis* could be explained by its high mobility and ability to move into the forest, thereby lowering exposure (Wanger et al., 2011). A lack of effects on the most common forest species (*H. celebensis*) might be due to limited exposure on the edges of cocoa plantations. In contrast, direct experimental exposure of amphibians to paraquat, cypermethrin, and the combination treatment negatively affected most species, including the common *I. celebensis*, under realistic field conditions. The difference of results between correlative and experimental field effect studies suggests that more such combined studies in particular with long term (>1 yr) correlative components are required to better understand the underlying reasons of such differences.

Glyphosate studies on reptiles are limited, being largely restricted to laboratory investigations (Latorre et al., 2013; Poletta et al., 2011), and we did not find any studies documenting the effect of paraquat on reptiles. Cypermethrin is known to cause genotoxicity, enzymatic and metabolic alterations, and growth delay in caimans (Poletta et al., 2011). As neither plot nor landscape variables showed a detectable effect on general reptile richness and abundance or *E. multifasciatus* abundance, perhaps because the concentrations were too low to affect this more resilient taxon with less permeable skin compared to amphibians (Weir et al., 2016). In contrast, in the experimental approach, paraquat and the combined paraquat/cypermethrin exposure reduced reptile abundance and richness, and eliminated common species. Less sensitive reptiles might move from areas with high to low pesticide load, again emphasizing the importance of landscape-level management. Cypermethrin also reduced reptile abundance and common species abundance. No effects on reptile richness from these chemicals could be the result of immigration of less sensitive species such as *Eutropis rudis* and *E.* cf. *grandis* into treated plots (Wanger et al., 2011), attracted by lower competition from absent more sensitive species.

Our results suggest that increasing pesticide use in our study region may decrease amphibian pest-control services. In previous work from the same study areas, we showed that *I. celebensis* can influence cocoa yield through a direct negative effect on invasive ants (*Anoplolepis gracilipes*) and an indirect positive effect on native ants (Wanger et al., 2010b). As invasive ants reduce cocoa yield by 34%, the common toads (*I. celebensis*) can act as an indirect cocoa pest control agents (Motzke et al., 2013; Wanger et al., 2010b). As commonly used pesticides eliminate these toads, but have both positive and negative effects on different ant species (Motzke et al., 2013), we speculate that farmers may lose amphibian pest-control services, and so paradoxically have more pests that they then may control with more pesticides (Fig. S5).

## Conclusions

In conclusion, our results show the strong effects that pesticides can have on both groups—under real field conditions. A better understanding of pesticide effects on tropical amphibians and reptiles is needed, because extrapolating existing results from temperate to tropical regions led to highly inconsistent results, and could only be based on freshwater and arthropod species (Daam & VandenBrink, 2010; Kwok et al., 2007; Daam et al., 2019). Moreover, regulations for pesticide registration in the United States and the EU do not yet require data on amphibians and reptiles (*e.g.*, EFSA et al., 2018). As tropical developing countries typically base their regulatory processes on those already in place in the United States or EU, pesticide-effect studies on amphibians and reptiles both in the temperate zone and in the tropics will help to protect these groups more effectively, in some of the most biodiverse regions on Earth.

##  Supplemental Information

10.7717/peerj.15046/supp-1 Supplemental Information 1 Supplementary Text, Tables, and Figures

10.7717/peerj.15046/supp-2 Supplemental Information 2 Raw Data

## Additional Information and Declarations

### Data Availability

The following information was supplied regarding data availability: The primary and meta-data on amphibian & reptile richness per treatment, pesticide load per treatment/ habitat type, habitat characteristics and a detailed species list are available in the Supplementary File.

## References

[ref-1] Agostini MG, Roesler I, Bonetto C, Ronco AE, Bilenca D (2020). Pesticides in the real world: the consequences of GMO-based intensive agriculture on native amphibians. Biological Conservation.

[ref-2] Annett R, Habibi HR, Hontela A (2014). Impact of glyphosate and glyphosate-based herbicides on the freshwater environment. Journal of Applied Toxicology.

[ref-3] Berger G, Graef F, Pfeffer H (2013). Glyphosate applications on arable fields considerably coincide with migrating amphibians. Scientific Reports.

[ref-4] Brodeur JC, Poliserpi MB, Sánchez M (2014). Synergy between glyphosate-and cypermethrin-based pesticides during acute exposures in tadpoles of the common South American Toad Rhinella arenarum. Chemosphere.

[ref-5] Brühl CA, Schmidt T, Pieper S, Alscher A (2013). Terrestrial pesticide exposure of amphibians: an underestimated cause of global decline?. Scientific Reports.

[ref-6] Bürkner PC (2017). Brms: an R package for Bayesian multilevel models using stan. Journal of Statistical Software.

[ref-7] Daam MA, Chelinho S, Niemeyer JC, Owojori OJ, De Silva PMCS, Sousa JP, van Gestel CAM, Römbke J (2019). Environmental risk assessment of pesticides in tropical terrestrial ecosystems: test procedures, current status and future perspectives. Ecotoxicology and Environmental Safety.

[ref-8] Daam MA, VandenBrink PJ (2010). Implications of differences between temperate and tropical freshwater ecosystems for the ecological risk assessment of pesticides. Ecotoxicology.

[ref-9] Ockleford C, Adriaanse P, Berny P, Brock T, Duquesne S, Grilli S, Hernandez-Jerez AF, Hougaard Bennekou S, Klein M, Kuhl T, Laskowski R, Machera K, Pelkonen O, Pieper S, Stemmer M, Sundh I, Teodorovic I, Tiktak A, Topping C, Wolterink G, Craig P, Jong F, Manachini B, Sousa P, Swarowsky K, Auteri D, Arena M, Smith RH, EFSA Panel on Plant Protection Products and their Residues (PPR) (2018). Scientific opinion on the state of the science on pesticide risk assessment for amphibians and reptiles. EFSA Journal.

[ref-10] Foley JA, Ramankutty N, Brauman KA, Cassidy ES, Gerber JS, Johnston M, Mueller ND, O’Connell C, Ray DK, West PC, Balzer C, Bennett EM, Carpenter SR, Hill J, Monfreda C, Polasky S, Rockstrom J, Sheehan J, Siebert S, Tilman D, Zaks DPM (2011). Solutions for a cultivated planet. Nature.

[ref-11] Fournier DA, Skaug HJ, Ancheta J, Ianelli J, Magnusson A, Maunder M, Nielsen A, Sibert J (2012). AD Model Builder: using automatic differentiation for statistical inference of highly parameterized complex nonlinear models. Optimal Methods Software.

[ref-12] Grant PB, Woudneh MB, Ross PS (2013). Pesticides in blood from spectacled caiman (Caiman crocodilus) downstream of banana plantations in Costa Rica. Environmental Toxicology and Chemistry.

[ref-13] Grass I, Loos J, Baensch S, Batáry P, Librán-Embid F, Ficiciyan A, Klaus F, Riechers M, Rosa J, Tiede J, Udy K, Westphal C, Wurz A, Tscharntke T (2019). Land-sharing/-sparing connectivity landscapes for ecosystem services and biodiversity conservation. People and Nature.

[ref-14] Hayes TB, Khoury V, Narayan A, Nazir M, Park A, Brown T, Adame L, Chan E, Buchholz D, Stueve T, Gallipeau S (2010). Atrazine induces complete feminization and chemical castration in male African clawed frogs (Xenopus laevis). Proceedings of the National Academy of Sciences of the United States of America.

[ref-15] Kremen C (2015). Reframing the land-sparing/land-sharing debate for biodiversity conservation. Annals of the New York Academy of Sciences.

[ref-16] Kwok KWH, Leung KMY, Lui GSG, Chu SVKH, Lam PKS, Morritt D, Maltby L, Brock T, Van den Brink PJ, Warne MJ, Crane M (2007). Comparison of tropical and temperate freshwater animal species’ acute sensitivities to chemicals: implications for deriving safe extrapolation factors. Integrated Environmental Assessment and Management.

[ref-17] Lanctôt C, Navarro-Martín L, Robertson C, Park B, Jackman P, Pauli BD, Trudeau VL (2014). Effects of glyphosate-based herbicides on survival, development, growth and sex ratios of wood frog (Lithobates sylvaticus) tadpoles. II: agriculturally relevant exposures to Roundup WeatherMax^®^ and Vision^®^ under laboratory conditions. Aquatic Toxicology.

[ref-18] Latorre MA, López González EC, Larriera A, Poletta GL, Siroski PA (2013). Effects of in vivo exposure to Roundup^®^ on immune system of Caiman latirostris. Journal of Immunotoxicology.

[ref-19] Mann RM, Hyne RV, Choung CB, Wilson SP (2009). Amphibians and agricultural chemicals: review of the risks in a complex environment. Environmental Pollution.

[ref-20] Motzke I, Tscharntke T, Sodhi NS, Klein A-M, Wanger TC (2013). Ant seed predation, pesticide applications and farmers’ income from tropical multi-cropping gardens. Agricultural and Forest Entomology.

[ref-21] Ortiz-Santaliestra ME, Maia JP, Egea-Serrano A, Brühl CA, Lopes I (2017). Biological relevance of the magnitude of effects (considering mortality, sub-lethal and reproductive effects) observed in studies with amphibians and reptiles in view of population level impacts on amphibians and reptiles. EFSA Supporting Publications.

[ref-22] Poletta GL, Kleinsorge E, Paonessa A, Mudry MD, Larriera A, Siroski PA (2011). Genetic, enzymatic and developmental alterations observed in Caiman latirostris exposed in ovo to pesticide formulations and mixtures in an experiment simulating environmental exposure. Ecotoxicology and Environmental Safety.

[ref-23] Quassinti L, Maccari E, Murri O, Bramucci M (2009). Effects of paraquat and glyphosate on steroidogenesis in gonads of the frog Rana esculenta in vitro. Pesticide Biochemistry and Physiology.

[ref-24] Relyea RA, Schoeppner NM, Hoverman JT (2005). Pesticides and amphibians: the importance of community context. Ecological Applications.

[ref-25] Sivula T, Magnusson M, Vehtari A (2020). Uncertainty in Bayesian leave-one-out cross-validation based model comparison.

[ref-26] Sturtz S, Ligges U, Gelman A (2005). R2WinBUGS: a package for running WinBUGS from R. Journal of Statistical Software.

[ref-27] Tamburini G, Bommarco R, Wanger TC, Kremen C, van der Heijden MGA, Liebman M, Hallin S (2020). Agricultural diversification promotes multiple ecosystem services without compromising yield. Science Advances.

[ref-28] Vehtari A, Gelman A, Gabry J (2017). Practical Bayesian model evaluation using leave-one-out cross-validation and WAIC. Statistics and Computing.

[ref-29] Wagner N, Reichenbecher W, Teichmann H, Tappeser B, Lötters S (2013). Questions concerning the potential impact of glyphosate-based herbicides on amphibians. Environmental Toxicology and Chemistry.

[ref-30] Wanger TC, De Clerck F, Garibaldi LA, Ghazoul J, Kleijn D, Klein AM, Kremen C, Mooney H, Perfecto I, Powell L, Settele J, Solé M, Tscharntke T, Weisser W (2020). Integrating agroecological production in a robust post-2020 global biodiversity framework. Nature Ecology & Evolution.

[ref-31] Wanger TC, Iskandar DT, Motzke I, Brook BW, Sodhi NS, Clough Y, Tscharntke T (2010a). Effects of land-use change on community composition of tropical amphibians and reptiles in Sulawesi, Indonesia. Conservation Biology.

[ref-32] Wanger TC, Motzke I, Saleh S, Iskandar DT (2011). The amphibians and reptiles of the Lore Lindu National Park area, Central Sulawesi, Indonesia. Salamandra.

[ref-33] Wanger TC, Rauf A, Schwarze S (2010). Pesticides and tropical biodiversity. Frontiers in Ecology and the Environment.

[ref-34] Wanger TC, Saro A, Iskandar DT, Brook BW, Sodhi NS, Clough Y, Tscharntke T (2009). Conservation value of cacao agroforestry for amphibians and reptiles in South-East Asia: combining correlative models with follow-up field experiments. Journal of Applied Ecology.

[ref-35] Wanger TC, Wielgoss AC, Motzke I, Clough Y, Brook BW, Sodhi NS, Tscharntke T (2010b). Endemic predators, invasive prey and native diversity. Proceedings of the Royal Society of London B: Biological Sciences.

[ref-36] Weir SM, Talent LG, Anderson TA, Salice CJ (2016). Insights into reptile dermal contaminant exposure: reptile skin permeability to pesticides. Chemosphere.

[ref-37] Yin XH, Li SN, Zhang L, Zhu GN, Zhuang HS (2008). Evaluation of DNA damage in Chinese toad (Bufo bufo gargarizans) after in vivo exposure to sublethal concentrations of four herbicides using the comet assay. Ecotoxicology.

[ref-38] Zuur AF, Ieno EN, Walker NJ, Saveliev AA, Smith GM (2009). Mixed effects models and extensions in ecology with R.

