# Supplementary material for: Pesticides reduce tropical amphibian and reptile diversity in agricultural landscapes in Indonesia
Source: PeerJ. 2023 Mar 20;11:e15046. doi: 10.7717/peerj.15046 (PMC10035417; doi:10.7717/peerj.15046)
Supplement: Supplemental Information 1 [file peerj-11-15046-s001.docx]

**Supplementary Material**

**Pesticides Reduce Tropical Amphibian and Reptile Diversity in Agricultural Landscapes in Indonesia**

Thomas C. Wanger^1,2*^, Barry W. Brook^3^, Theodore Evans^4,5^, Teja Tscharntke^2*^

^1^ Sustainable Agricultural Systems & Engineering Laboratory, Westlake University, Hangzhou, China

^2^ Agroecology, University of Göttingen, Göttingen, Germany

^3^ School of Biological Sciences, University of Tasmania, Hobart, Australia

^4^ Department of Biological Sciences, National University of Singapore, Singapore, Singapore ^5^ School of Biological Sciences, The University of Western Australia, Perth, Australia

***Correspondence:**

Thomas C. Wanger ([tomcwanger@gmail.com](mailto:tomcwanger@gmail.com)) & Teja Tscharntke (ttschar@gwdg.de)

**Supplementary Text T1 - Pesticide-Use Questionnaire Details**

Site owners were asked whether they give their voluntary consent to participate in the interviews. If they gave their consent, which all plot owners did, they were asked for the pesticide brand used and the number of pesticide container caps applied per month per plot. We then asked the owner to show us the original container and checked (1) the amount of pesticide per cap of the container; (2) the amount of caps / 10 liters of water as indicated in the manual; and (3) dosage instructions. Based on these values, we calculated the amount of pesticide applied per plot per year.

**Supplementary Table S1** **– Details of sampled habitats**

Summary of characteristics of all sampled habitats. *Shrub Volume* = shrub density × shrub height; *Number of trees* = number of trees in a plot with a diameter at breast height ≥ 50 cm. Values are means (SD) by habitat. *NS-cacao agroforest* = natural-shaded cacao agroforest; *PS-cacao agroforest* = planted-shaded cacao agroforest. Variables are as described in the [Supplementary Material](http://onlinelibrary.wiley.com/doi/10.1111/j.1523-1739.2009.01434.x/suppinfo) of Wanger et al. (2010A).

|  | **Canopy Cover [%]** | **Annual pesticide use [ml/plot]** | **Leaf-litter cover [%]** | **Leaf-litter thickness [cm]** | **No. logs** | **Shrub Volume** | **No. trees** | **Canopy heterogeneity** |
| --- | --- | --- | --- | --- | --- | --- | --- | --- |
| **Primary forest** | 94.9 (1.2) | 0.0 (0.0) | 72.5 (27.6) | 2.1 (1.0) | 6.2 (2.7) | 408.7 (228.0) | 11.5 (6.0) | 2.5 (0.3) |
| **Secondary forest** | 87.0 (2.2) | 21.4 (56.7) | 70.3 (20.9) | 2.4 (0.7) | 9.4 (3.8) | 370.8 (252.3) | 9.0 (4.0) | 2.7 (0.5) |
| **NS-cacao agroforest** | 77.8 (4.4) | 81.4 (103.5) | 37.3 (19.5) | 1.7 (0.9) | 6.3 (4.7) | 289.5 (217.7) | 3.9 (3.5) | 3.1 (0.3) |
| **PS-cacao agroforest** | 66.0 (3.8) | 145.0 (136.1) | 29.0 (25.2) | 1.0 (0.6) | 6.7 (5.1) | 240.9 (240.3) | 2.7 (2.6) | 2.6 (0.5) |
| **Open areas** | 0.0 (0.0) | 384.0 (411.0) | 0.0 (0.0) | 0.0 (0.0) | 3.2 (1.6) | 61.0 (24.9) | 0.0 (0.0) | 1.2 (0.0) |

**Supplementary Table S2a – Encountered amphibian species.** Red List classifications follow the criteria of IUCN (IUCN 2019, http://www.iucnredlist.org): CE = Critically Endangered; E = Endangered; V = Vulnerable; NT = Near Threatened; LC = Least Concern; DD =Data Deficient; NE = not evaluated by the IUCN; Specialist [Y/N/?] = species considered a pristine-forest specialist/disturbance-tolerant species/not known; Habitat encountered = habitat where we encountered the species during the three-year survey; abbreviations used: PF = Rainforest; SF = secondary forest; AF = Cacao agroforest; CP = Cacao plantation; OA = Open area; RF = Rice paddy. Abundance was classified as C = commonly encountered; R = rare (as mentioned in the literature).

| **Amphibian species** | **IUCN Red List** | **Specialist** | **Habitat encountered** | **Abundance** |
| --- | --- | --- | --- | --- |
| **Bufonidae** |  |  |  |  |
| *Duttaphrynus melanosticus* | LC | N | AF, CP, HS | C |
| *Ingerophrynus celebensis* | LC | N | PF, SF, AF, CP, OA | C |
| **Microhylidae** |  |  |  |  |
| *Kaloula pulchra* | LC | N | PF | C |
| *Oreophryne* sp. 1 | NE | N | PF, SF | R |
| **Ranidae** |  |  |  |  |
| *Hylarana celebensis* | LC | N | PF, SF | C |
| **Dicroglossidae** |  |  |  |  |
| *Fejervarya limnocharis* | LC | N | PF, SF, AF, CP, OA, RF | C |
| *Limnonectes* cf. *arathooni* | NE | Y | PF | R |
| *Limnonectes* cf. *modestus* | NE | N | PF, SF | R |
| *Limnonectes* cf. *heinrichi* | NE | Y | AF | R |
| *Limnonectes* sp. (medium 3) | NE | Y | PF | R |

**Supplementary Table S2b** **– Encountered reptile species.** Abbreviations are the same as in Tab. S2a

| **Reptile Species** | **IUCN Red List** | **Specialist** | **Habitat encountered** | **Abundance** |
| --- | --- | --- | --- | --- |
| **Colubridae** |  |  |  |  |
| *Ahaetulla prasina* | NE | N | SF, AF, CP | C |
| *Boiga irregularis* | NE | N | AF | C |
| *Dendrelaphis pictus pictus* | NE | N | AF, CP | C |
| **Natricidae** |  |  |  |  |
| *Rhabdophis callistus* | NE | N | SF | R |
| **Lamprophiidae** |  |  |  |  |
| *Psammodynastes pulverulentus pulverulentus* | NE | N | SF | C |
| **Xenopeltidae** |  |  |  |  |
| *Xenopeltis unicolor* | NE | N | AF, CP | C |
| **Agamidae** |  |  |  |  |
| *Bronchocela celebensis* | NE | N | PF, SF,AF, CP | C |
| **Gekkonidae** |  |  |  |  |
| *Cyrtodactylus jellesmae* | NE | Y | SF, AF | R |
| **Scincidae** |  |  |  |  |
| *Eutropis* sp. | NE | N | SF, AF, CP, OA | C |
| *Eutropis multifasciatus* | NE | Y | CP, OA | C |
| *Eutropis rudis* | NE | N | PF, SF, AF, CP | C |
| *Parvoscincus* sp. | NE | ? | PF, SF, AF, CP | ? |
| *Sphenomorphus* cf. *textus* | NE | ? | PF, AF, CP, OA | ? |
| *Sphenomorphus nigrilabris* | NE | Y | PF, SF, AF, CP | ? |
| *Sphenomorphus cf. variegatus* | NE | N | PF, SF, AF, OA | ? |

**Supplementary Note S1. Details on the Chemical Substances used in this Study**

***Paraquat:*** “Noxon” Brand; CAS number 1910-42-5; a.i. content 297 g l^-1^ / viologen redox-active herbicide / mode of action: interferes with the electron transport chain in photosynthesis

***Theta-cypermethrin:*** “Tetrin” Brand; CAS number 71697-59-1; a.i. content 30 g l^-1^ / synthetic pyrethroid neurotoxic insecticide / mode of action: interferes with sodium channels in nerve axons.


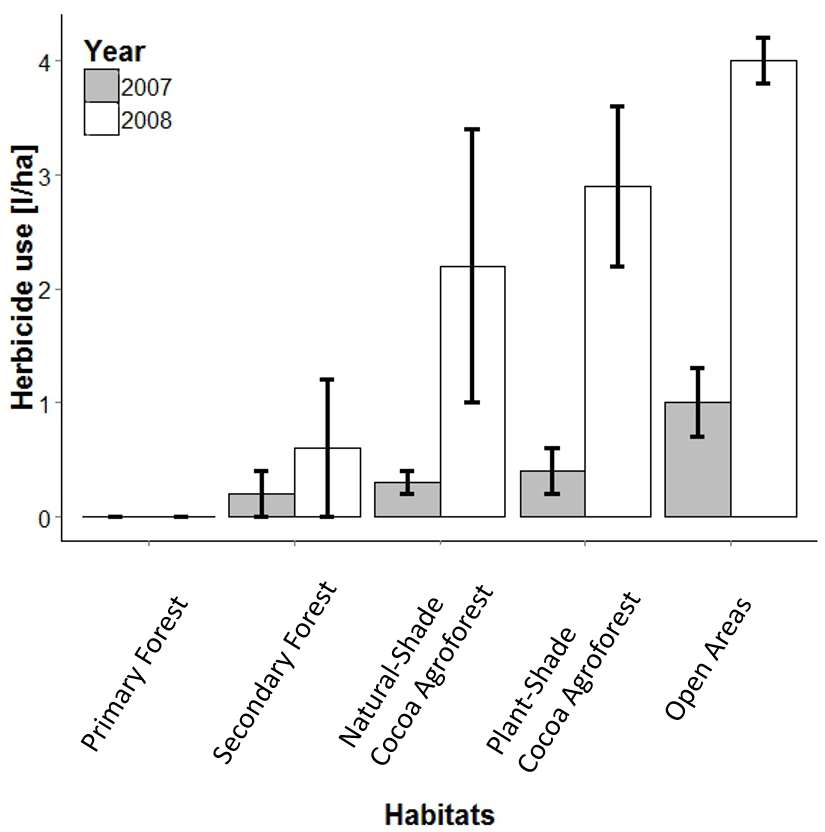


**Supplementary Figure S1.** Herbicide use (mean ± standard error) across five habitat types during the correlative long-term approach. *Natural-Shade Cocoa Agroforest* are cocoa plantations with natural forest shade trees opposed to *Plant-Shade Cocoa Agroforest*, where shade comes from planted trees. The variation in pesticide use in secondary forest might be a result of some plots being prepared for cocoa production in 2008. In cocoa plantations, farmer’s perception of pesticide prices and their income has the potential to modify application patterns heavily. Pesticide use was uniformly high in open areas that have also seen the strongest increase in pesticide use from 1 to 4 l/ha from 2007 to 2008

**
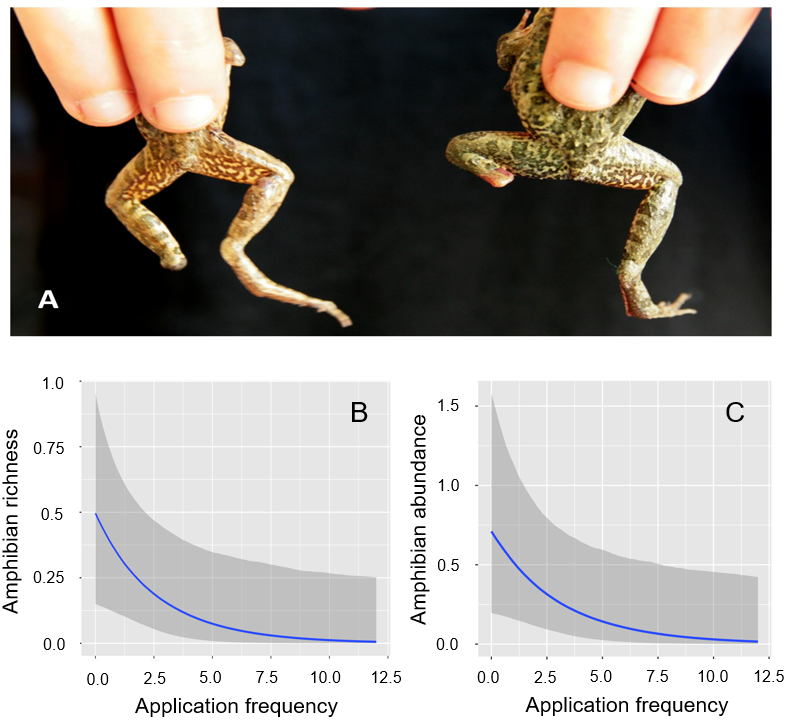
Supplementary Figure S2.** Pesticide effects on a common amphibian species and general amphibian and reptile abundance. We found several Fejervarya limnocharis with deformed limbs in the heavily sprayed sites of the study region (A). As confirmed by the modelling approach presented in Tab. 1 and Tab. 2, increasing pesticide application frequency negatively affected amphibian richness (B) and abundance. Reported abundances are on the plot level.


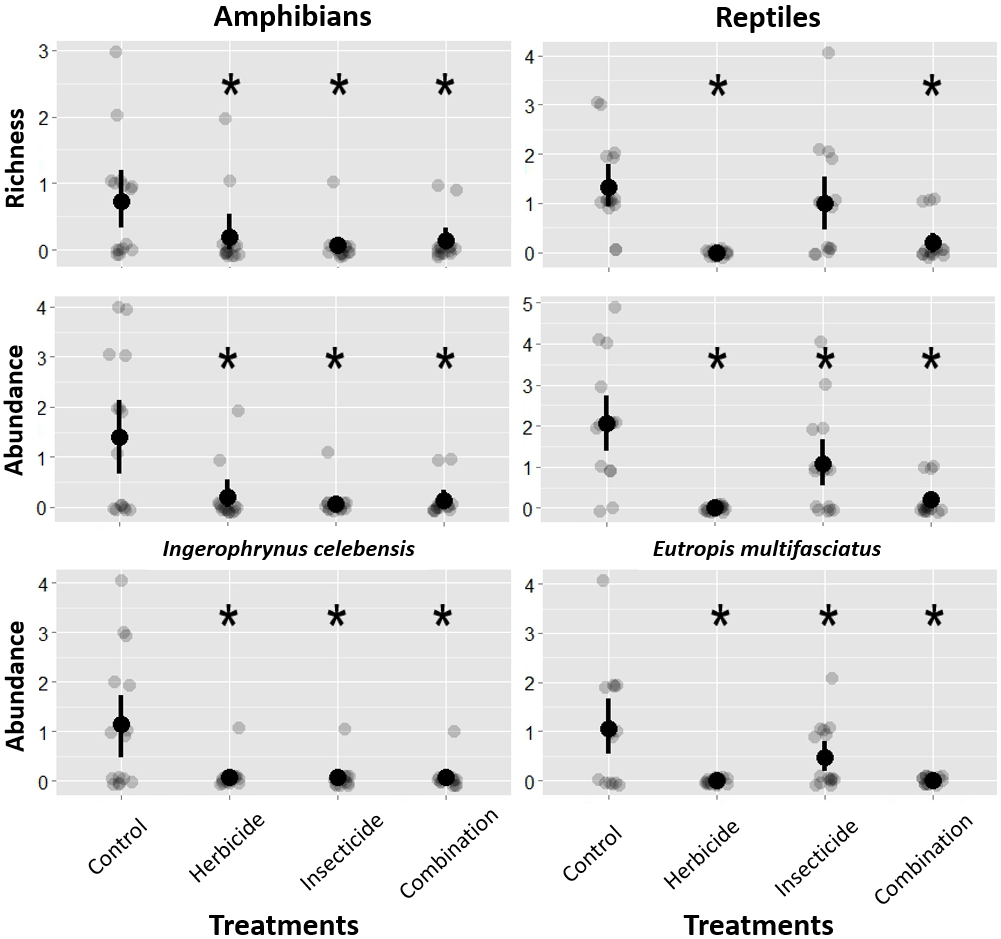


**Supplementary Fig. S3.** Effects of experimental pesticide treatments on the species richness and abundance of all amphibians and reptiles, and separately for abundance of the two most common species in both taxa (bottom row). Stars indicate an effect that is measurably different from the control (for calculated treatment effects see Fig. 1). Black dot and lines indicate mean and standard errors, respectively. The light grey dots represent the individual data points jittered by the factor 0.1 to enhance clarity.

­­­
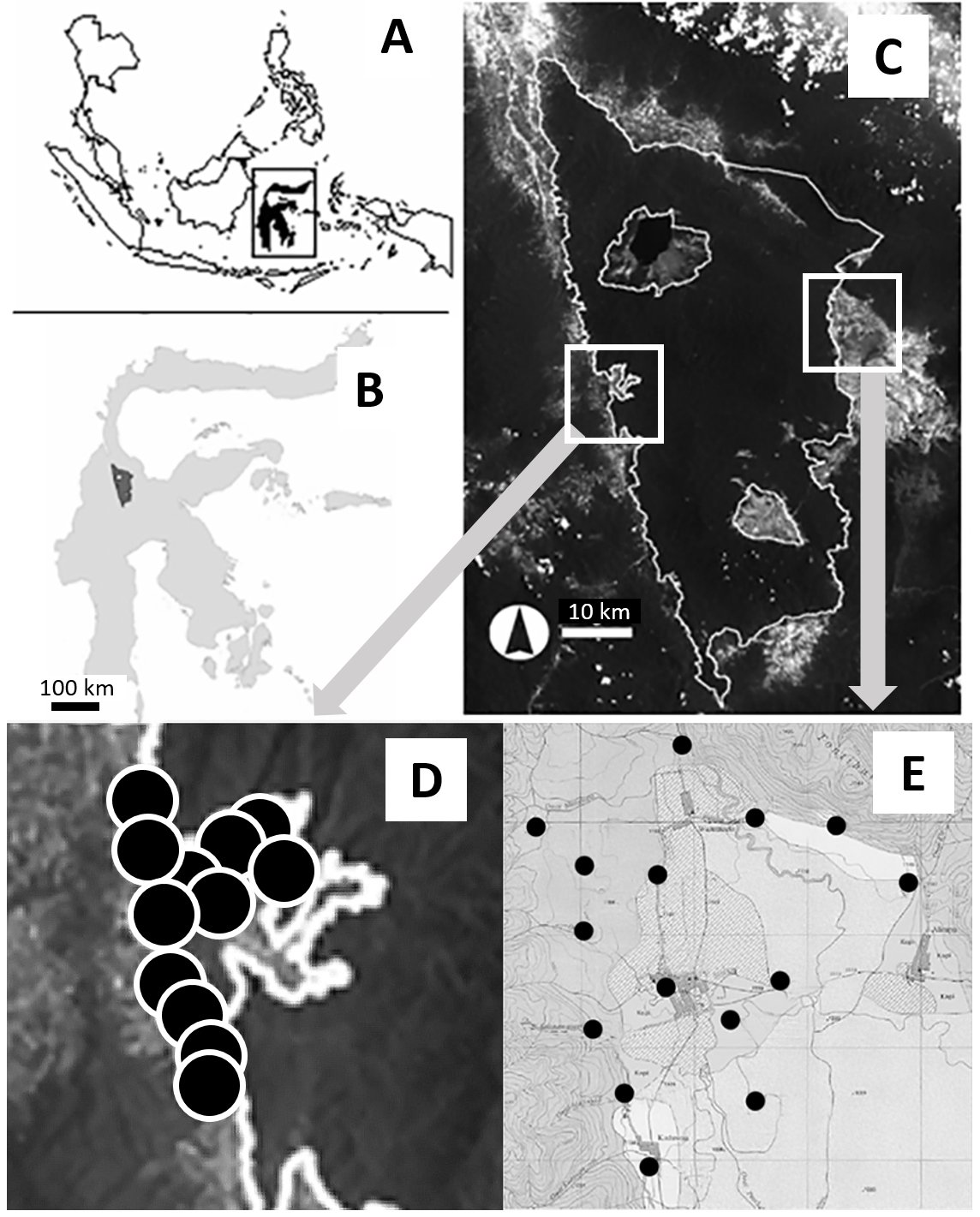


**Supplementary Fig. S4.** Plot locations in Indonesia (A) on Sulawesi (B) around the Lore Lindu National Park (C). Shown are the correlative sites (D) and the experimental sites (modified from Wanger et al. 2009 and Motzke et al. 2013). Note that only a subset of the correlative sites is shown due to missing GPS coordinates.


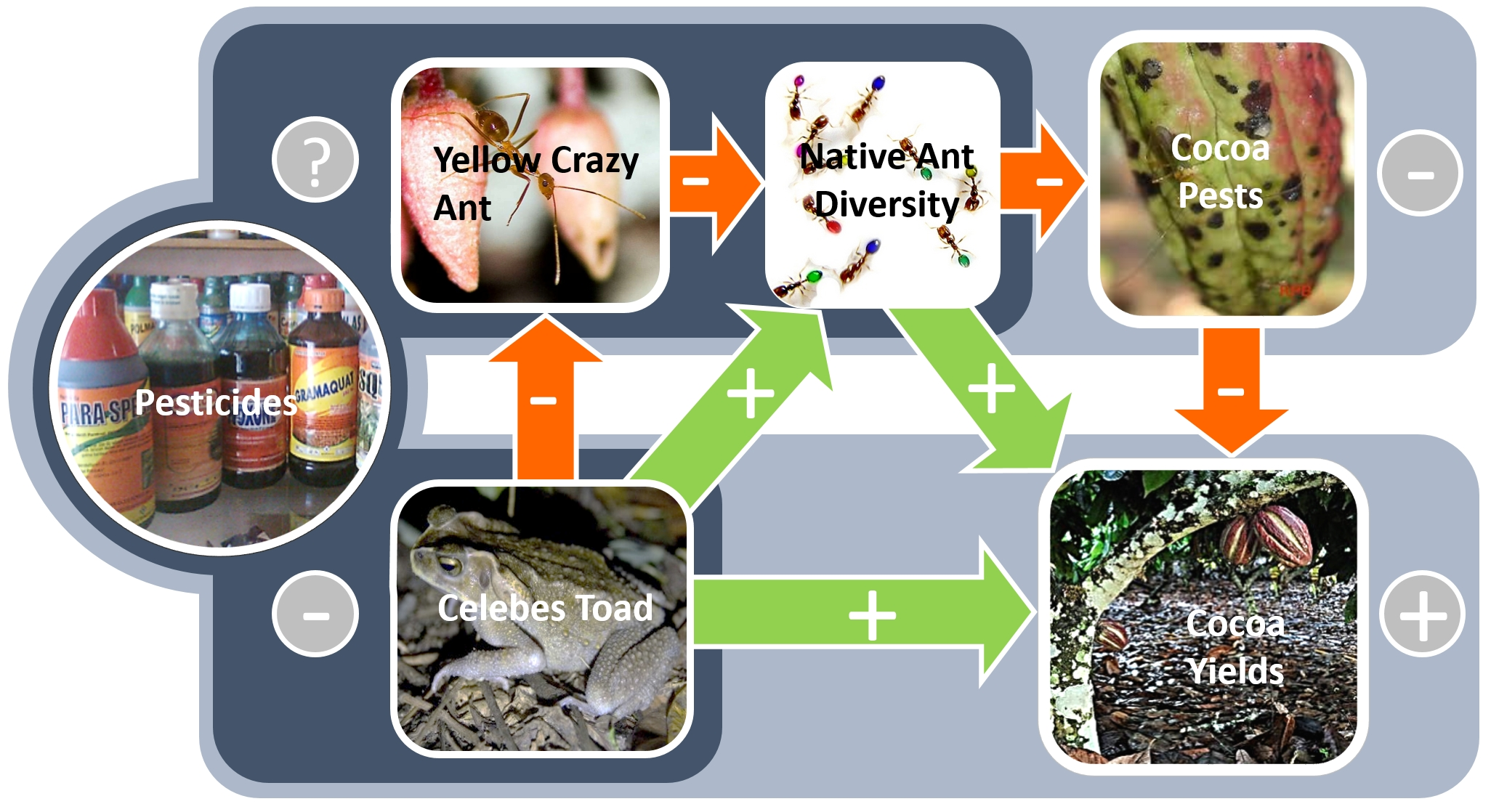


**Supplementary Fig. S5.** Indirect pesticide effects on pest-control-service provisioning by an endemic toad in cocoa agroforestry landscapes. The toads exert a negative pressure on the interaction between invasive Yellow Crazy ants and native ant diversity, which leads to a decrease in pest damage on cocoa and therefore enhances crop yields. The toad’s indirect positive effects on cocoa productivity are, however, suppressed (dark blue areas) by pesticide use, which has uniform effects on invasive ants and native ant diversity. Thus although yields will benefit from intentional effects (light blue areas) of pesticide use, farmers will also lose free pest-control ‘ecosystem services’, which may reduce pesticide expenses (see text for more details; Wanger et al. 2010B).
